# Identification of a 1,2,4-Oxadiazole with Potent and Specific Activity against *Clostridioides difficile*, the Causative Bacterium of *C. difficile* Infection, an Urgent Public Health Threat

**DOI:** 10.1021/acs.jmedchem.3c01778

**Published:** 2023-10-12

**Authors:** Michael R. Barbachyn

## Abstract

The discovery of compound **57**, a new, totally synthetic 1,2,4-oxadiazole antibacterial agent, is described. This oxadiazole displays highly selective, bactericidal killing of *Clostridioides (Clostridium) difficile*, the bacterium that causes *C. difficile* infection (CDI) in both hospital and community settings. The narrow spectrum of activity exhibited by **57** should avoid any disruption of commensal anaerobic bacteria in the gut microbiome, minimizing chances for recurrent CDI.

## Introduction

*Clostridioides difficile*, formerly known as *Clostridium difficile*, is a toxin-producing, Gram-positive anaerobic bacterium that causes colitis (inflammation of the colon) and diarrhea in both hospital and community settings. *C. difficile* infection (CDI) has been the most common healthcare-associated bacterial infection for many years and was identified by the Centers for Disease Control and Prevention (CDC) as an urgent public health threat in 2013, and again in 2019.^1^ As might be expected, the COVID-19 pandemic caused a significant disruption in CDI surveillance data collection from 2020 onward, but the most comprehensive recent case reports from 2019 remain disturbing. It is estimated that there were 202,600 hospital cases of CDI in the United States in 2019, with 11,500 deaths.^2^ The morbidity and mortality associated with CDI are largely the result of *C. difficile*’s production of toxins A (TcdA) and B (TcdB), which cause the observed colitis, diarrhea, and dehydration.^3^

The recommended first-line therapeutic options for treating CDI include orally administered vancomycin or fidaxomicin (metronidazole is no longer recommended when vancomycin or fidaxomicin is available).^4^ Fortunately, *C. difficile* resistance to vancomycin and fidaxomicin is reported only rarely.^5^ Unfortunately, all available CDI antibacterial agents suffer from three primary deficiencies: 1) varying degrees of disruption of the normal gut microbiome, 2) recurrent CDI (rCDI) in roughly 25% of patients within several months of discontinuation of therapy, and 3) a lack of activity against *C. difficile* spore germination, which contributes to rCDI.^3^ Recurrent CDI is associated with a significantly higher risk of downstream death. This recurrence phenomenon is a unique aspect of *C. difficile*, resulting from its existence in both active (vegetative) and dormant (spore) forms. Marketed antibacterial agents only target the vegetative form of *C. difficile*, leaving the spores to eventually germinate and reactivate CDI. Because of the above-noted limitations, there exists a real need for the identification of new treatment modalities that address some or all of these real-world shortcomings.

## Discussion

**Figure 1:**
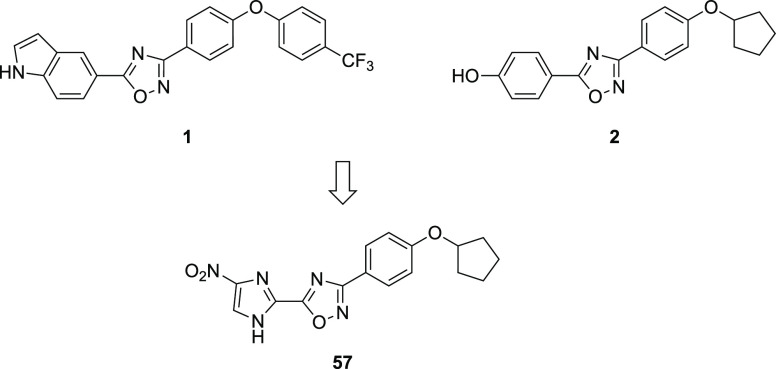
Progenitor anti-staphylococcal 1,2,4-oxadiazole compounds **1** and **2** and anti-*Clostridioides difficile* nitroimidazole-substituted lead compound **57**.

In this issue, Qian and co-workers report the identification of a new, totally synthetic antibacterial agent which, minimally, inhibits cell-wall peptidoglycan biosynthesis in *C. difficile*.^6^ This project had its genesis in earlier seminal work reported by the Mobashery lab wherein in silico screening led to the identification of the novel anti-staphylococcal 1,2,4-oxadiazole class of penicillin-binding protein (PBP) 2a inhibitors.^7^ The lead oxadiazole identified at that time, compound **1** (Figure 1), exhibited good in vitro activity against methicillin-resistant *Staphylococcus aureus* (MRSA), along with commensurate in vivo efficacy in murine MRSA infection models. Subsequently, Janardhanan and co-workers reported the interesting phenol derivative **2**, which also exhibits good anti-staphylococcal activity.^8^ Fortuitously, both **1** and **2** were also found to have good in vitro activity against *C. difficile* ATCC 43255, with minimum inhibitory concentrations (MICs) of 4 and 2 μg/mL, respectively.^8^ After screening the team’s existing library of 75 oxadiazoles for activity against *C. difficile* ATCC 43255, some preliminary structure–activity relationships (SARs) were identified for this series that led to the syntheses of an additional 58 analogs. The culmination of this effort was the identification of compound **57**, a potent and very selective 1,2,4-oxadiazole derivative with a MIC of 0.25 μg/mL against *C. difficile* ATCC 43255. Compound **57** is the primary focus of the current work.

**Figure 2:**
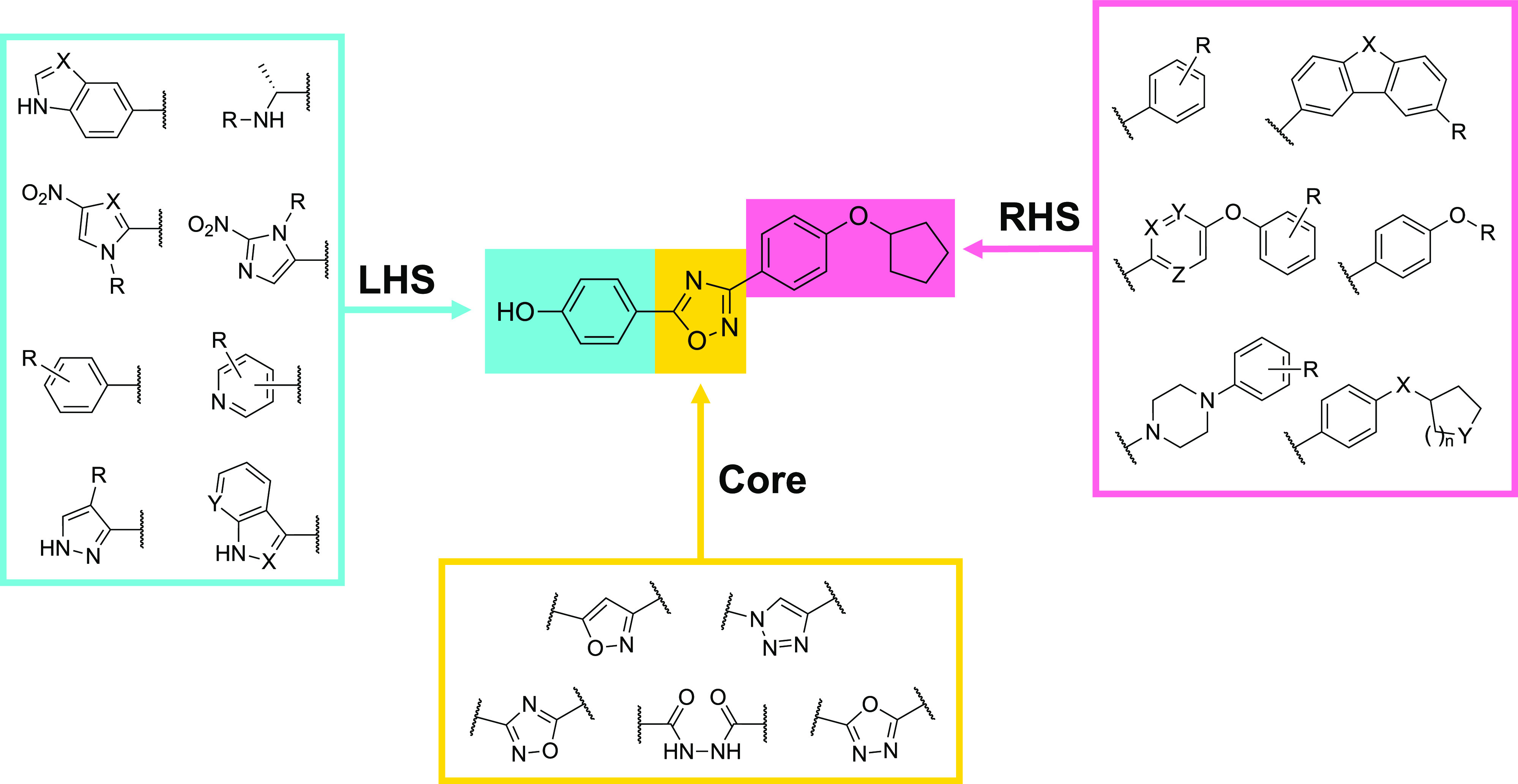
Modular strategy and targets identified for profiling as next-generation oxadiazole analogs.

Figure 2 presents a high-level summary of the oxadiazole derivatives targeted in this campaign, with compound **2** being the starting point for conceptual changes to the overall structure. The design plan looks to have been largely empirical, with a heavy focus on isosteric replacements. Both the left-hand side (LHS) and right-hand side (RHS) were systematically modified, as shown in the figure. Finally, the central 1,2,4-oxadiazole core was also investigated via the incorporation of various acyclic and cyclic subunits. The targeted 1,2,4-oxadiazole antibacterial agents were rapidly assembled via an efficient three-step, modular process passing through a key amidoxime intermediate.^6^ Condensation/cyclization to give the central oxadiazole ring system was achieved by reacting an activated carboxylic acid with the amidoxime under one of three sets of reaction conditions.

The synthesized oxadiazole analogs (133 total) were initially screened for activity against *C. difficile* ATCC 43255. Compound **57** quickly emerged as one of the most potent compounds in the oxadiazole collection (MIC = 0.25 μg/mL). This compound was also shown to be rapidly bactericidal against *C. difficile* in time–kill studies and developed resistance much more slowly than the marketed comparator vancomycin. A scanning electron microscopy (SEM) study of vegetative *C. difficile* treated with **57** indicated significant damage to the bacterial cell wall, consistent with the expected 1,2,4-oxadiazole mode of action (MOA) involving inhibition of peptidoglycan biosynthesis. Analog **57** also exhibited an in vitro therapeutic index of >512 in a relevant cytotoxicity screen (lactate dehydrogenase assay with THP-1 human monocyte cells), suggesting good selectivity for prokaryotic organisms such as *C. difficile*. It should be noted that the aromatic nitro group of **57** is a structural alert and may eventually require further investigation into possible isosteric replacements. On the other hand, this functional group may be perfectly acceptable for an antibiotic focused on intestinal infections, where oral delivery, high fecal concentrations, and (hopefully) low oral bioavailability are typically the norm.

An evaluation of oxadiazole **57** against an expanded panel of both aerobic and anaerobic bacteria was conducted. This extensive study revealed a remarkably selective and unique spectrum of antibacterial activity for compound **57**. Importantly, the lead compound exhibited very good activity against both sensitive and multi-drug-resistant (MDR) strains (*n* = 101) of *C. difficile*, with a MIC_90_ of 1 μg/mL. Oxadiazole **57** was totally inactive against aerobic Gram-positive and Gram-negative bacteria. This is in contrast to the results obtained for its progenitor oxadiazoles **1** and **2**, which exhibited good activity against Gram-positive aerobes such as the staphylococci, streptococci, and enterococci. The extremely narrow spectrum of activity for oxadiazole **57**, encompassing just *C. difficile*, was further supported by its total lack of activity against other common anaerobic gut bacteria. No data was provided to clarify whether **57** retains the ability to inhibit *C. difficile* spore germination, as was demonstrated both in vitro and in vivo for its predecessor, oxadiazole **2**.^8^ Obviously, prevention of spore germination would further help prevent rCDI.

**Figure 3:**
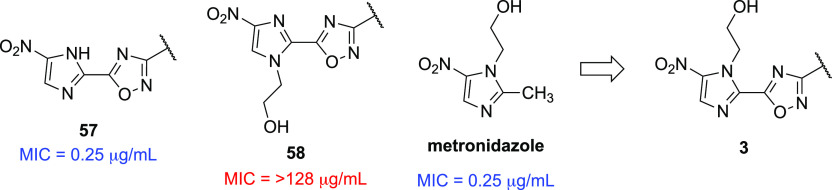
Nitroimidazole derivatives **57**, **58**, metronidazole, and analog **3**.

The reasons for the unprecedented, *C. difficile*-specific activity of **57** remain unclear at this point and will require additional investigation. Certainly, **57** was shown (SEM study) to inhibit cell wall biosynthesis, suggesting that it is an inhibitor of PBP and peptidoglycan biosynthesis. Perhaps **57** expresses a unique selectivity for one of the *C. difficile* PBPs? Perhaps more likely is the possibility that oxadiazole **57** exhibits more than one MOA. Metronidazole (see Figure 3), as at least part of its MOA, relies on cellular reductases to reduce its nitro group to a nitroso radical, which then goes on to inhibit DNA synthesis and repair. This begs the question as to whether **57**, which bears some structural resemblance to metronidazole, is also a substrate for these reductases and if this reactive manifold might contribute to the compound’s overall antibacterial effect(s). The study under discussion did examine the hydroxyethyl-substituted congener **58**, which was found to be totally inactive (MIC = > 128 μg/mL), as well as a regioisomeric *N*-methylimidazole analog (compound **59** in ref (6)), which exhibited a weak MIC of 32 μg/mL. However, the direct oxadiazole comparator to metronidazole, regioisomer **3**, was not described. Regardless, more investigation is needed to further clarify the MOA of oxadiazole **57**. Some insights into the MOA of **57** might be gleaned from its study in a macromolecular synthesis assay, where the incorporation of radiolabeled precursors is monitored. This would help identify the biosynthesis pathway(s) (cell wall synthesis, protein synthesis, DNA synthesis, RNA synthesis, etc.) being inhibited by **57** in a concentration-dependent manner. In addition, does **57** also exhibit the ability to inhibit *C. difficile* spore germination, like its parent oxadiazole **2**, and so reduce the likelihood of rCDI?^8^ No indication of this capability was provided in the work under discussion.

## Conclusion

Building on the past discovery of the totally synthetic 1,2,4-oxadiazole class of antibacterial agents, which target PBP 2a in Gram-positive pathogens, the work in ref (6) reports the identification of a structurally new oxadiazole, compound **57**, with remarkable selectivity for the anaerobe *C. difficile*, the causative bacterium of CDI, currently designated as the most urgent bacterial threat in United States hospitals.^1,2^ The importance of this pre-clinical agent should not be underestimated. Despite the availability of marketed agents such as vancomycin and, more recently, fidaxomicin, there remains an urgent need for new treatment regimens utilizing a unique MOA that will avoid target-based cross-resistance with other antibiotics. An oxadiazole exclusively targeting *C. difficile* has the potential to avoid disturbing/killing commensal gut bacteria, which contributes to *C. difficile* overgrowth and rCDI. It is also hoped that compound **57**, like its progenitor **2**, will inhibit *C. difficile* spore germination and so help break the cycle of rCDI that plagues patients suffering from this medical condition.

## References

[ref1] Centers for Disease Control and Prevention. Antibiotic Resistance Threats in the United States, 2019; U.S. Department of Health and Human Services: Atlanta, GA, 2019. 10.15620/cdc:82532.

[ref2] Centers for Disease Control and Prevention. COVID-19: U.S. Impact on Antimicrobial Resistance, Special Report 2022; U.S. Department of Health and Human Services: Atlanta, GA, 2022. 10.15620/cdc:117915.

[ref3] Jarmo O.; Veli-Jukka A.; Eero M. Treatment of Clostridioides (Clostridium) difficile infection. Ann. Med. 2020, 52, 12–20. 10.1080/07853890.2019.1701703. 31801387 PMC7877971

[ref4] Van Prehn J.; Reigadas E.; Vogelzang E. H.; Bouza E.; Hristea A.; Guery B.; Krutova M.; Noren T.; Allerberger F.; Coia J. E.; Goorhuis A.; van Rossen T. M.; Ooijevaar R. E.; Burns K.; Scharvik Olesen B. R.; Tschudin-Sutter S.; Wilcox M. H.; Vehreschild M. J. G. T.; Fitzpatrick F.; Kuijper E. J. European Society of Clinical Microbiology and Infectious Diseases: 2021 update on the treatment guidance document for *Clostridioides difficile* infection in adults. Clin. Microbiol. Inf. 2021, 27, S1–S21. 10.1016/j.cmi.2021.09.038. 34678515

[ref5] Sholeh M.; Krutova M.; Forouzesh M.; Mironov S.; Sadeghifard N.; Molaeipour L.; Maleki A.; Kouhsari E. Antimicrobial resistance in *Clostridioides (Clostridium) difficile* derived from humans: a systematic review and meta-analysis. Antimicrob. Resist. Infect. Control 2020, 9, 1–11. 10.1186/s13756-020-00815-5. 32977835 PMC7517813

[ref6] Qian Y.; Birhanu B. T.; Yang J.; Ding D.; Janardhanan J.; Mobashery S.; Chang M. A Potent and Narrow-Spectrum Antibacterial Against *Clostridioides difficile* Infection. J. Med. Chem. 2023, 10.1021/acs.jmedchem.3c01249. PMC11681498 37732641

[ref7] aO’Daniel P. I.; Peng Z.; Pi H.; Testero S. A.; Ding D.; Spink E.; Leemans E.; Boudreau M. A.; Yamaguchi T.; Schroeder V. A.; Wolter W. R.; Llarrull L. I.; Song W.; Lastochkin E.; Kumarasiri M.; Antunes N. T.; Espahbodi M.; Lichtenwalter K.; Suckow M. A.; Vakulenko S.; Mobashery S.; Chang M. Discovery of a new class of non-beta-lactam inhibitors of penicillin-binding proteins with Gram-positive antibacterial activity. J. Am. Chem. Soc. 2014, 136, 3664–3672. 10.1021/ja500053x. 24517363 PMC3985699

[ref8] Janardhanan J.; Kim C.; Qian Y.; Yang J.; Meisel J. E.; Ding D.; Speri E.; Schroeder V. A.; Wolter W. R.; Oliver A. G.; Mobashery S.; Chang M. A dual-action antibiotic that kills Clostridioides difficile vegetative cells and inhibits spore germination. Proc. Natl. Acad. Sci. U.S.A. 2023, 120, e2304110120 10.1073/pnas.2304110120. 37155891 PMC10193928

